# Counterfactual prediction from machine learning models: transportability and joint analysis for model development and evaluation using multi-source data

**DOI:** 10.1186/s41512-025-00201-y

**Published:** 2025-10-02

**Authors:** Sarah C. Voter, Issa J. Dahabreh, Christopher B. Boyer, Habib Rahbar, Despina Kontos, Jon A. Steingrimsson

**Affiliations:** 1 Department of Biostatistics, Brown University School of Public Health, Providence, RI USA; 2 CAUSALab, Harvard T.H. Chan School of Public Health, Boston, MA USA; 3 Department of Epidemiology, Harvard T.H. Chan School of Public Health, Boston, MA USA; 4 Department of Biostatistics, Harvard T.H. Chan School of Public Health, Boston, MA USA; 5 Department of Radiology, University of Washington, Seattle, WA USA; 6 Department of Radiology, University of Pennsylvania, Philadelphia, PA USA

**Keywords:** Machine learning, Model evaluation, Transportability, Observational analysis, Counterfactual prediction

## Abstract

**Background:**

When a machine learning model is developed and evaluated in a setting where the treatment assignment process differs from the setting of intended model deployment, failure to account for this difference can lead to suboptimal model development and biased estimates of model performance.

**Methods:**

We consider the setting where data from a randomized trial and an observational study emulating the trial are available for machine learning model development and evaluation. We provide two approaches for estimating the model and assessing model performance under a hypothetical treatment strategy in the target population underlying the observational study. The first approach uses counterfactual predictions from the observational study only and relies on the assumption of conditional exchangeability between treated and untreated individuals (no unmeasured confounding). The second approach leverages the exchangeability between treatment groups in the trial (supported by study design) to “transport” estimates from the trial to the population underlying the observational study, relying on an additional assumption of conditional exchangeability between the populations underlying the observational study and the randomized trial.

**Results:**

We examine the assumptions underlying both approaches for fitting the model and estimating performance in the target population and provide estimators for both objectives. We then develop a joint estimation strategy that combines data from the trial and the observational study, and discuss benchmarking of the trial and observational results.

**Conclusions:**

Both the observational and transportability analyses can be used to fit a model and estimate performance under a counterfactual treatment strategy in the population underlying the observational data, but they rely on different assumptions. In either case, the assumptions are untestable, and deciding which method is more appropriate requires careful contextual consideration. If all assumptions hold, then combining the data from the observational study and the randomized trial can be used for more efficient estimation.

**Supplementary information:**

The online version contains supplementary material available at 10.1186/s41512-025-00201-y.

## Introduction

Users of machine learning models are often interested in adapting and/or evaluating the model in the target population where the model derived predictions are intended to be used in. But machine learning models are often fit and/or evaluated on data that differs from the target population in terms of treatments used post baseline and/or the distribution of covariates. In such scenarios, fitting a model and evaluating model performance in the target population involves counterfactual questions under hypothetical scenarios, hereafter referred to as counterfactual predictions [1–5]. Compared to factual predictions that only depend on observed variables and do not involve hypothetical “what if” questions, counterfactual predictions are more challenging to evaluate and require stronger assumptions [5, 6]. With the rising use of machine learning models in healthcare settings, such “what-if” questions arise naturally in the context of patient risk prediction under possible treatment regimes. We consider the setting where the observational database for which we would like to perform counterfactual prediction on, is accompanied by a randomized trial that shares the same eligibility criteria, treatment, outcome measures and has an overlapping set of baseline covariates [7–9]. Randomized trials almost always enroll rather than randomly sample participants leading to a convenience sample that might not be representative of the desired target population [10–13]. However, observational databases such as electronic health records or medical claims are sometimes thought to be more representative and provide “real world” data on diverse set of participants in routine clinical care [14–16]. The use of more representative datasources can help with addressing fairness concerns, a topic of increasing importance as machine learning models are deployed in a variety of settings [17]. The under-representation of marginalized groups in clinical trials has been well documented [18–20]. This lack of diversity, given known differences in comorbidity profiles and treatment response within these populations can limit the predictive ability of machine learning models built using trial data [21, 22]. Thus, throughout we focus on drawing inferences about the population underlying the sample in the observational study (i.e., the target population).

Recently, several methods have been developed for counterfactual predictions and evaluation of model performance under hypothetical treatment strategies from observational data [3, 6, 23–25]. A key assumption underpinning such analyses is the assumption of conditional exchangeability of treated and untreated participants within levels of baseline covariates. This assumption is not only untestable, but likely often violated in observational studies, particularly when treatment decisions are affected by variables that are difficult to measure. A potential remedy for this would be to incorporate information from a randomized controlled trial where the conditional exchangeability assumption is supported by randomization of treatment assignment. However, interpreting the analysis of randomized trial data in the context of the target population requires accounting for potential differences between the populations underlying the randomized trial and the target population [26, 27]. Such transportability analyses rely on the different assumption of exchangeability between the population underlying the randomized trial and the observational study conditional on baseline covariates. In this manuscript, we contrast the two approaches and discuss benchmarking of the results (i.e., comparisons of the estimates from the observational and transportability analysis). We furthermore provide assumptions under which joint analysis of the two datasources is valid and derive estimators and properties of estimators for jointly analyzing the two data sources. We provide an illustration of the estimators and use them to estimate the performance of a random forest model on the Coronary Artery Surgery Study (CASS) under hypothetical treatment strategies.

## Data structure and objectives

Suppose we have data from an observational study and a randomized trial. For both data sources, we have data on a fully observed (i.e., uncensored) outcome $Y$, a binary treatment assignment $A$ (randomized in the trial but not in the observational study), and a baseline covariate vector $X$. We focus on the setting where there is complete adherence to treatment assignment and no missing data. Denote participation in the randomized trial by $S$ (i.e., $S=1$ for observations that are from the randomized trial and $S=0$ for observations that are from the observational study). Let $n_0$ be the sample size of the observational study, $n_1$ be the sample size of the randomized trial, and $n = n_0 + n_1$ be the sample size in the combined dataset. We denote by $Y^a$ the potential outcome under the intervention to set treatment to $A=a$ [28, 29].

Here, we assume a non-nested sampling design [30] where the data in the observational study and the randomized trial are sampled separately from their underlying super-populations with unknown and likely unequal sampling probabilities. Results for the nested design can be found in the Appendix. Although we model the data from the randomized trial as coming from some super-population (that might be ill-defined), we do not assume that the sample is obtained through a formal sampling process. However, we assume that the data from the observational study is a representative sample from a population of clinical relevance.

Our objective is to build a machine learning model for the conditional expectation of the potential outcome under the treatment strategy that everyone receives treatment $A=a$ where $a \in \{0,1\}$ in the target population and evaluate the performance of a model in the target population under this same treatment strategy. In the Appendix, we present results for more general treatment strategies.

## Assumptions

Now we will present and discuss two different sets of identifiability assumptions. The first approach, which we refer to as the observational analysis, relies on the following assumptions.

- A1: Consistency in the observational study. For all individuals *i* with $S_i=0$, we have $Y_i^a = Y_i$ if $A_i=a$.
- A2: Conditional exchangeability between treatment groups in the population underlying the observational study ($Y^a \perp \!\!\!\perp A|X,S=0$).
- A3: Positivity of treatment assignment in the population underlying the observational study. That is for $a \in \{0,1\}$ and for all covariate patterns that can occur in the population underlying the observational study, there is a non-zero probability of receiving treatment *a*.

The consistency assumption A1 implies: (i) no interference (i.e., the potential outcomes of one participant are not influenced by other participants), (ii) variation in how the treatment is administered does not affect outcomes [31] (i.e., no hidden versions of treatment), and (iii) study participation only affects outcomes through treatment assignment (i.e., there are no Hawthorne effects). The conditional exchangeability between treatment groups assumption (A2) is often referred to as the “no unmeasured confounding” assumption, as it implies that there are no unmeasured variables that affect both treatment assignment and the outcome (which is supported by design when treatment is randomized, but is an untestable assumption in any observational analysis). The positivity of treatment assignment assumption says that all individuals should have a positive probability of receiving all treatments (which also holds by design in randomized trials).

Now, suppose that we suspect considerable violation of the conditional exchangeability assumption (A2) in the observational study, then an alternative is to “transport” results obtained from the randomized trial to the target population underlying the observational study. Identifiability of the transportability approach relies on the following assumptions:

- A1*: Consistency in the randomized trial and the observational study. For all individuals *i*, we have $Y_i^a = Y_i$ if $A_i=a$.
- A2*: Conditional exchangeability between treatment groups in the randomized trial ($Y^a \perp \!\!\!\perp A|X,S=1$).
- A3*: Positivity of treatment assignment in the randomized trial. That is for $a \in \{0,1\}$ and for all covariate patterns that can occur in the population underlying the randomized trial, there is a non-zero probability of receiving treatment *a*.
- A4*: Conditional exchangeability between populations underlying the randomized trial and the observational study ($Y^a \perp \!\!\!\perp S|X$).
- A5*: Positivity of being in the target population. For all covariate patterns that can occur in the population underlying the observational study, there is a non-zero probability of the covariate pattern occurring in the randomized trial.

Assumptions A2* and A3* are supported by design in randomized trials. The conditional exchangeability between population assumption (A4*) implies that the measured covariates $X$ are enough to account for between population differences. The positivity of being in the target population assumption (A5*) says that the randomized trial has at least as broad of a spectrum as the observational study, but it does allow the distribution of the covariates to be different between the randomized trial and the observational study. This means that transportability analysis is not feasible in cases where the covariate distribution of the observational study spans regions of ineligibility for the randomized trial (unless the investigator willing to rely on untestable extrapolation assumptions). The positivity assumptions A3* and A5* can be examined using the observed data, but assessing their validity can be challenging [32].

## Fitting a model for counterfactual predictions in the target population

Now we show how observational analysis and transportability analysis can be used to fit a machine learning model for the conditional expectation of the potential outcome mean under a counterfactual treatment strategy in the target population underlying the observational study. In other words, we derive results for identifiability of the estimand $\mu _a(X^*) \equiv \textrm{E}[Y^a|X^*,S=0]$ and associated estimation procedures. We refer to a model that has been built to estimate $\textrm{E}[Y^a|X^*,S=0]$ as a tailored model. As $\mu _a(X^*)$ depends on the unknown potential outcome $Y^a$, it is not a function of the observed data. Here, $X^*$ is a subset of $X$ , the set of covariates required for conditional exchangeable assumptions *A*2, *A*2* and *A*4*. For instance, many common clinical prediction tools used by physicians are based on a small number of easy-to-obtain measurements ($X^*$), but a more high dimensional covariate vector might be required for assumptions *A*2 and *A*4* to hold $\left(X\right)$.

### Observational analysis

If assumptions *A*1 through *A*3 hold, we can write $\mu _a(X^*) = \textrm{E} [\textrm{E}[Y | X, S=0,A=a] |X^* , S=0]$. The appearance of iterated expectations in the expression above suggests a two-step estimation strategy similar to the procedure described in [23]. The first step is to fit a model for $Y$ conditional on the full set of covariates $X$ among the subset of participants in the observational study with treatment $A=a$. Next, as described by Boyer et al. [23], the second expectation can either be estimated non-parametrically when the number of covariates in $X^*$ is small, or in higher-dimensional cases, by regressing the predicted values from the first model on the subset of covariates $X^*$ among all participants in the observational database ($S=0$). In the Appendix, we provide an alternative inverse weighting identifiability expression and the associated inverse weighting estimator.

### Transportability analysis

If assumptions A1* through A5* hold, then an alternative way to write $\mu _a(X^*)$ is through the transportability identifiability result $\mu _a(X^*) = \textrm{E} [\textrm{E}[Y | X, S=1,A=a] |X^* , S=0]$. Similarly to the observational analysis this result suggests a two-step estimation strategy where in first step a model for $Y$ conditional on the full set of covariates $X$ is fit using data from participants assigned to treatment $A=a$ in the randomized trial and the second step can be implemented as the second step for the observational analysis.

### Joint analysis

If assumptions *A*1 through *A*3 and *A*1* through *A*5* hold, then $E[Y | X, S=1,A=a] = E[Y | X, S=0,A=a] = E[Y|X,A=a]$, suggesting the following “joint analysis” identifiability result $\mu _a(X^*) = \textrm{E} [\textrm{E}[Y | X, A=a] |X^* , S=0]$. This identifiability result suggests fitting a model for $\textrm{E}[Y | X, A=a]$ using the pooled data from the randomized trial and the observational database and then regressing the predictions from that model on $X^*$ among participants in the observational database. In the Appendix, we provide an alternative inverse weighting expression and associated inverse weighting estimator.

### Inference

For any of the estimators described above, at a fixed value of $X^*$, standard error estimates are obtainable using resampling methods or the Huber-White sandwich estimator in the case of the two-step least-squares parametric estimation procedure [33–35]. For uniform inference across a range of low dimensional $X^*$ values, one can obtain uniform confidence bands using the weighted bootstrap procedure detailed in [36]. For cases of high-dimensional $X^*$, it may be difficult or computationally infeasible to construct a comprehensive grid capturing all relevant covariate patterns. In these cases, it may be useful to apply high-dimensional random sampling methods such as Latin Hypercube sampling [37], or to select a small subset of covariate patterns of clinical relevance.

## Estimating performance of a machine learning model in the target population

### Identifiability

Throughout this section we do not make the assumption that the model is correctly specified or that the model is tailored to a particular treatment strategy. To emphasize this, we focus on estimating model performance of an arbitrary model $g(X^*)$. Let $L(Y^a, g(X^*))$ denote a generic loss function that compares the potential outcome $Y^a$ with the predicted value $g(X^*)$. Common examples include the mean squared error, Brier loss, and absolute loss. Our target parameter, the quantity we want to estimate, is the expected loss (risk) in the target population $(S=0)$ under counterfactual treatment strategy $A =a$. That is, the target parameter is $\psi (a) \equiv \textrm{E} \left[ L(Y^a, g(X^*)) \mid S=0\right]$. This depends on the potential outcome $Y^a$ which for each observation is unobserved and hence $\textrm{E}\left[ L(Y^a, g(X^*)) \mid S=0\right]$ is not a function of the observed data. If assumptions A1 through A3 hold, then the counterfactual risk in the target population can be written as the observed data functional

1 $$\begin{aligned} \psi _{obs}(a) = \textrm{E}[ \textrm{E}[ L(Y, g(X^*)) |X, S=0, A=a] |S=0]. \end{aligned}$$

A derivation of this result is provided in the Appendix (also shown in [23]).

If assumptions A1* through A5* hold, then the counterfactual risk in the target population can be written as as the observed data functional

2 $$\begin{aligned} \psi _{tr}(a) = \textrm{E}[ E[ L(Y, g(X^*)) |X, S=1, A=a] |S=0]. \end{aligned}$$

For completeness, a derivation of this result is provided in the Appendix (also shown in [26]). Note that expressions (1) and (2) only rely on observed data (i.e., they do not involve counterfactual outcomes). Also, expression (2) only involves the distribution of the outcome conditional on covariates and treatment assignment in the randomized trial and the marginal covariate distribution in the observational database. Thus, it does not rely on outcome or treatment information from the observational database, which can be a benefit when outcome information is not available from the observational data or is unusable (e.g., due to few events or gross measurement error).

### Estimation

The identifiability results for the observational and transportability analysis suggest two estimators that are constructed as sample analogs of expressions (1) and (2). For the observational analysis this can be done using the following steps: (i) estimate $\textrm{E}[L(Y, g(X^*)) |X, S=0, A=a]$ using the data from the observational study, (ii) use this estimator to create predictions for $\textrm{E}[L(Y, g(X^*)) |X, S=0, A=a]$ for each covariate pattern (*X*) observed in the observational study, and (iii) average these predictions to get the estimator from the observational analysis. Mathematically, the estimator from the observational analysis is expressed as $\widehat{\psi }_{obs}(a) = \frac{1}{n_0} \sum _{i=1}^n I(S_i=0) \widehat{h}_{a,0}(X_i)$, where $\widehat{h}_{a,s}(X)$ is an estimator for $\textrm{E}[L(Y, g(X^*)) |X, S=s, A=a]$. Such estimators are often referred to as *outcome model estimators* [23, 27] to reflect that they fit a model for the conditional distribution of the outcome they wish to estimate ($\widehat{h}_{a,s}(X)$). Similarly for the transportability analysis, the outcome model estimator is obtained by (i) estimate $\textrm{E}[L(Y, g(X^*)) |X, S=1, A=a]$ using the data from the randomized trial, (ii) use the estimator to create predictions for $\textrm{E}[L(Y, g(X^*)) |X, S=1, A=a]$ for each covariate vector (*X*) in the observational study, and (iii) average these predictions to get the estimator from the transportability analysis. Mathematically, the estimator is expressed as $\widehat{\psi }_{tr}(a) = \frac{1}{n_0} \sum _{i=1}^n I(S_i=0 ) \widehat{h}_{a,1}(X_i).$

Note that for both the observational and transportability analyses, our general procedure is to obtain a counterfactual treatment-specific outcome model (*step (i)*), which is then used to obtain predictions in the observational study (*steps (ii) *and *(iii)*). In both cases, steps (ii) and (iii) are performed on the observational data because our target parameter, the counterfactual treatment model performance, is defined with regard to the population underlying the observational study. The difference between the two methods lies in that the observational analysis estimates the outcome model using the observational data and the transportability analysis uses the randomized trial data. In the Appendix, we present alternative doubly robust estimators for both the observational and the transportability analysis that are more robust to the modeling assumptions made.

## Illustrative example

Now we illustrate the concepts using a simple simulation with the outcome model estimators. We simulate a continuous outcome $Y$, binary treatment $A$, and one-dimensional continuous covariate vectors $X$ (measured) and $U$ (unmeasured). The data-generating mechanism is structured such that we can selectively violate the conditional exchangeability assumptions A2, and/or A4* (all other assumptions are satisfied in this simulation setting). In this example, we focus on counterfactual prediction under treatment $a=1$ using the mean squared error (MSE) as the measure of model performance. In the Appendix we present more details on how the data was simulated.

**Fig. 1:**
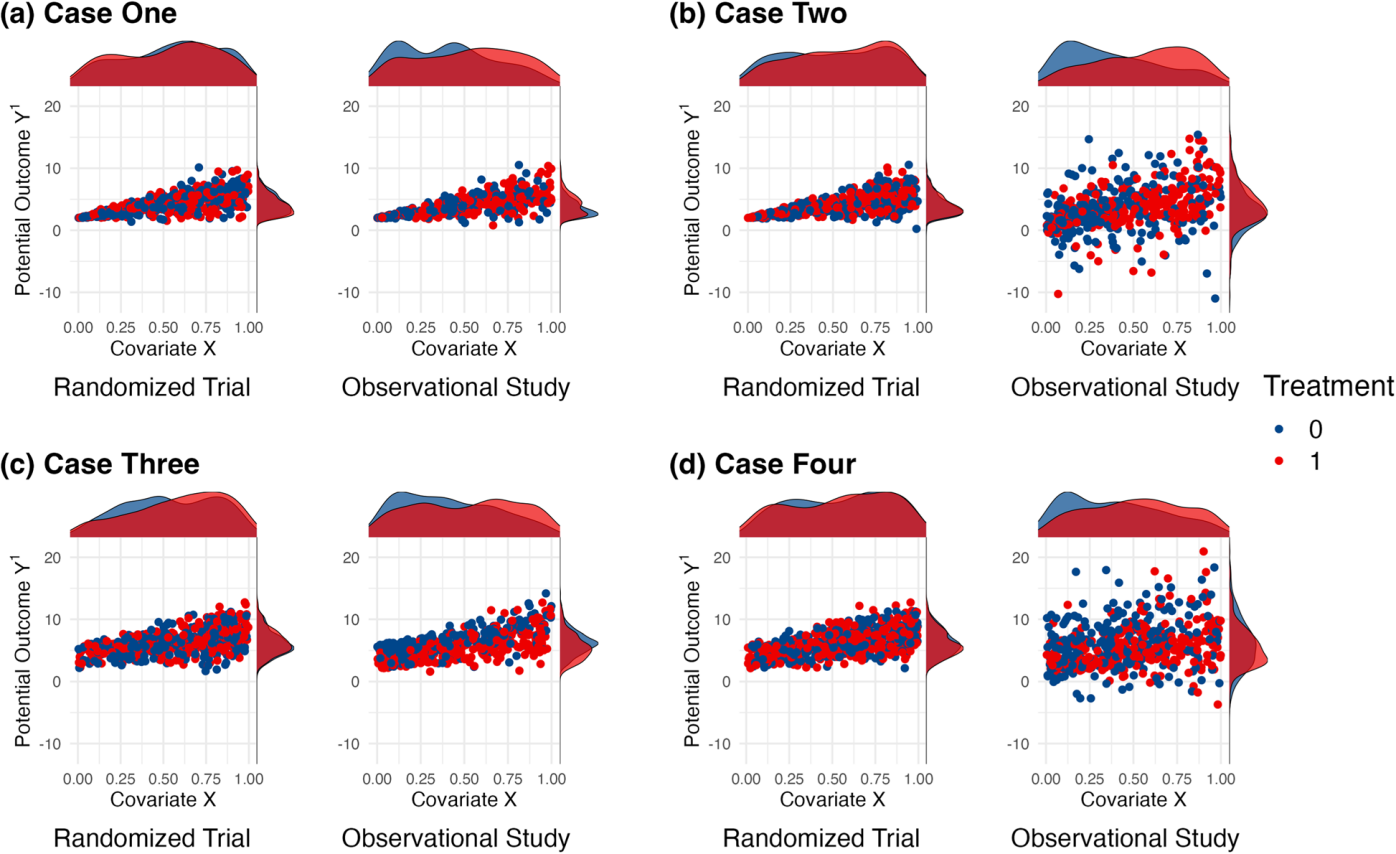
Visual examples of data representing each of the four cases described in this illustration. Each plot shows scatterplots of the counterfactual outcome $Y^1$ vs. the observed cova﻿ri﻿ate $X$. The density of $Y^1$ by treatment group is shown on the right of each plot and the density of $X$ by treatment group is shown above each plot

**Fig. 2:**
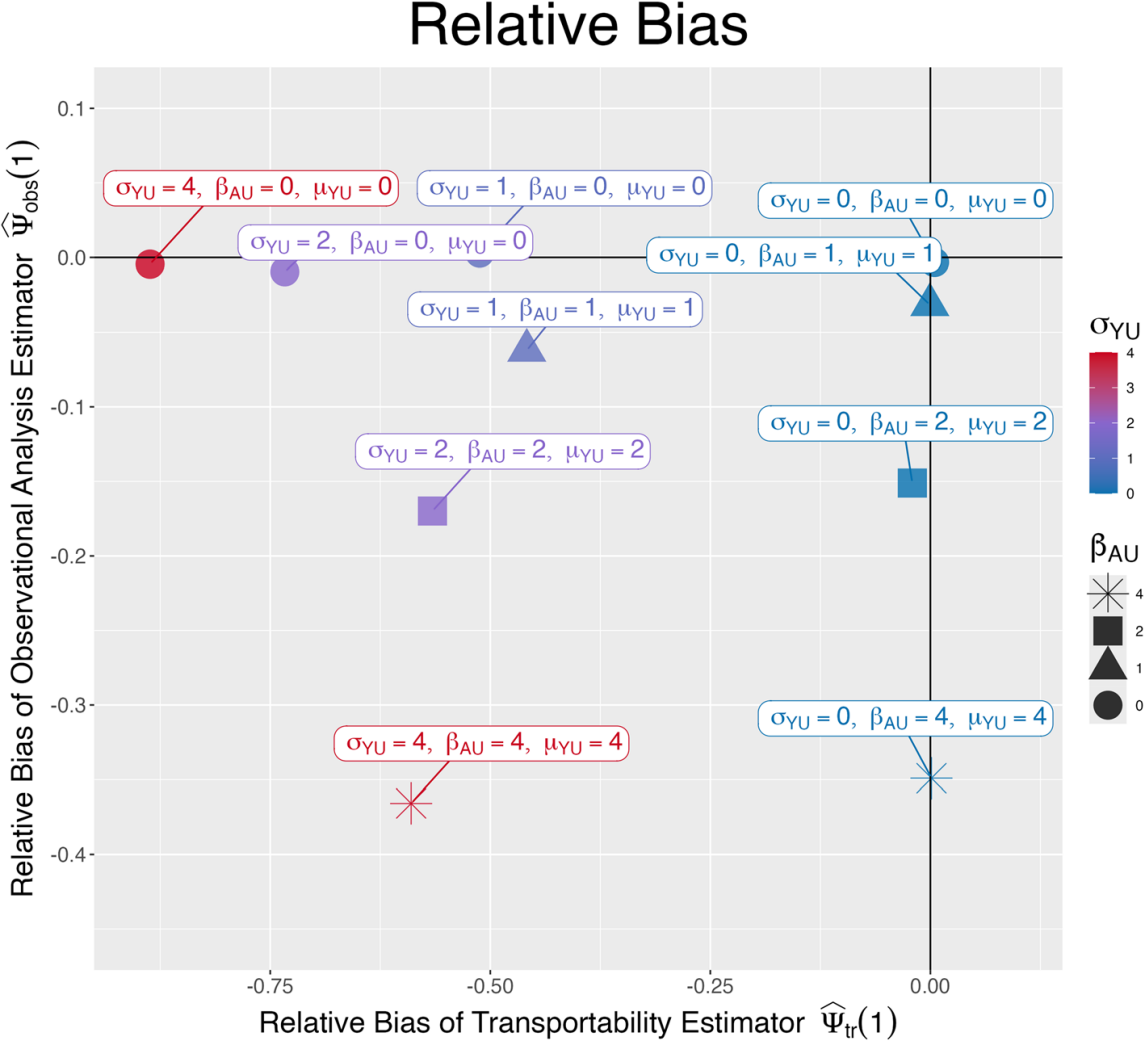
Relative bias of the observational estimate $\widehat{\psi }_{obs}(1)$ plotted against the bias of the transportability estimate $\widehat{\psi }_{tr}(1)$ when estimating the counterfactual mean in the population underlying the observational study if everyone was assigned to treatment $A=1$. If $\beta _{AU}>0$ and $\mu _{YU}>0$, then assumption A2 is violated and we expect the estimator from the observational analysis $\widehat{\psi }_{obs}(1)$ to be biased. If $\sigma _{YU}>0$, then assumption A4* is violated and we expect the estimator from the transportability analysis $\widehat{\psi }_{tr}(1)$ to be biased

To selectively violate assumption A2 without violating assumption A4*, we simulate $U$ as an unmeasured confounder that affects the potential outcome $Y^1$ in both the randomized trial and the observational study, but $U$ affects treatment assignment $A$ only in the observational study. We make $Y^1$ depend on $U$ by setting the parameter $\mu _{YU}>0$ and we make $A$ depend on $U$ by setting the parameter $\beta _{AU}> 0$. Thus, in our setup $\mu _{YU}>0$ and $\beta _{AU}>0$ imply that the estimator from the observational analysis is biased. To violate assumption A4* we make the MSE of $Y^1$ depend $U$ only in the population underlying the observational study through the parameter $\sigma _{YU}$ ($\sigma _{YU}>0$ implies violations of assumption A4*). Figures 1 and 2 illustrate and present, respectively, results from four different cases that differ in what assumptions are violated.

***Case 1****: Both estimators are unbiased (*$\beta _{AU} = \mu _{YU} = \sigma _{YU}=0$*)*. An example of such a dataset is shown in Fig. 1a and the figure shows that within levels of $X$ the variability in $Y^1$ is the same in both populations (assumption A4* holds). Figure 1a also shows that in the observational study treatment assignme $A$ is not predictive of the potential outcome $Y^1$ within levels of $X$ (assumption A2 holds). Figure 1 in the Appendix shows the relationship between $Y^1$ and the unmeasured covariate $U$ for the four cases considered in the main text. The simulation results, averaged across 500 simulations, under these conditions corresponds to a point close to the origin of the plot in Fig. 2, showing that both estimators are unbiased.

***Case 2****: Transportability estimator biased and observational estimator unbiased (*$\sigma _{YU}>0; \quad \beta _{AU} = \mu _{YU} = 0$*).* Figure 1b shows a simulated dataset from this setting. Her, $X$ alone is not sufficient to adjust for differences between the populations underlying the randomized trial and the observational study, as the unmeasured covariate introduces variability in $Y^1$ within levels of $X$ in a way that the variability is much larger in the observational study than in the randomized trial. This leads to the estimator from the transportability analysis underestimating the MSE in the population underlying the observational study. But as condition A2 holds, the estimator from the observational analysis is unbiased. As expected, the points for which $\sigma _{YU}$ is the only non-zero parameter appear in the upper-left quadrant of Fig. 2, corresponding to the observational estimator being unbiased and the transportability estimator having a negative bias that increases as $\sigma _{YU}$ increases.

***Case 3****: Transportability estimator unbiased and observational estimator biased (*$\beta _{AU}, \mu _{YU}> 0; \quad \sigma _{YU}=0$*).* In this case, the assumption of conditional exchangeability between populations (A4*) holds. But conditional exchangeability between treatment groups in the observational study is violated (A2) as in the observational study, even conditional on $X$, treatment assignment $A$ is informative about the potential outcome $Y^1$ (i.e., observations with $A=1$ generally have lower values of $Y^1$ than observations with $A=0$ with the same *X* value). This results in bias of the observational estimator (in the Appendix, we provide further details on how it is violated). The results in Fig. 2 show that when $\beta _{AU}, \mu _{YU}> 0$ and $\sigma _{YU}=0$ then the transportability estimator is unbiased and the observational estimator is biased.

***Case 4****: Both estimators biased (*$\sigma _{YU}, \beta _{AU} \mu _{YU}> 0$*).* In this scenario, both A2 and A4* are violated by making all three parameters non-zero. This leads to bias in both estimators, and the corresponding points in Fig. 2 appear in the lower lefthand quadrant.

### Benchmarking and joint analysis

Following [38], we define benchmarking as comparing the results from the analysis of the randomized trial and the observational study. Successful benchmarking (i.e., concordant results from the observational and transportability analysis) likely increase the trust in the analysis, but it does not guarantee validity as some assumptions (most likely either A2 and A4*) could be violated in a way such that the observational and the transportability estimators are both biased with a bias of similar magnitude and in the same direction.

Observational databases are often substantially larger than randomized trials allowing for more fine grained analysis than is possible with smaller datasets (e.g., subset analysis or analysis of rare outcomes). Thus, successful benchmarking could be used to support analysis of observational data that is infeasible using data from the randomized trial. If benchmarking is not successful, then it suggests that at least one assumption is not satisfied (likely one or both of A2 or A4*) but it cannot be inferred from the data which assumption is violated [38].

One way to determine whether the observational estimator and transportability estimator are concordant is to construct confidence intervals of their difference (e.g., using the non-parametric bootstrap). Such determination should also involve subject matter knowledge including the clinical significance of the magnitude of the differences between the two point estimates. If the observational estimator and transportability estimator are concordant and subject matter knowledge does not suggest violations of any of the identifiability assumptions, then a natural question is whether and how the data from the randomized trial and the observational study can be combined for more efficient estimation of the counterfactual risk in the target population[38–40].

One approach for joint analysis is to use some weighted combination of $\widehat{\psi }_{tr}$, and $\widehat{\psi }_{obs}$ (e.g, using equal weights, weights proportional to the sample size, and the inverse of the estimator specific variance). An alternative approach is based on the observation that if assumptions A1 through A3 and A1* through A5* hold, then

3 $$\begin{aligned} \textrm{E}[L(Y, g(X^*)) |X, &S=1, A=a] = \textrm{E}[L(Y, g(X^*)) |X, \\&S=0, A=a] = \textrm{E}[L(Y, g(X^*)) |X, A=a]. \end{aligned}$$

The equalities in expression (3) only rely on observed data distributions and are therefore testable using the observed data [41], but when $X$ is high dimensional conducting such tests can be challenging.

Using Eq. (3) we can write the counterfactual risk in the target population as $\psi _{joint}(a) = \textrm{E}[\textrm{E}[L(Y, g(X^*)) |X, A=a]| S=0]$ and the corresponding estimator that combines data from both datasets is $\widehat{\psi }_{joint} = \frac{1}{n_0} \sum _{i=1}^n I(S_i=0 ) \widehat{h}_{a}(X_i).$ Here, $\widehat{h}_{a}(X)$ is an estimator for $\textrm{E}[L(Y, g(X^*)) |X, A=a]$ estimated using the combined data from the randomized trial and the observational study. In the Appendix, we derive a doubly robust estimator for the counterfactual risk in the target population that combines data from the randomized trial and the observational study.

If all identifiability assumptions hold and with appropriately chosen estimators for the nuisance functions needed for their implementation, then the estimators obtained from an observational analysis, transportability analysis, and the joint analysis are unbiased and asymptotically normal. Hence, comparing the asymptotic variance of the three estimators is a natural thing to consider when choosing between them. The joint analysis relies on more assumptions that allows the estimator to use more data than both the estimators from the observational and the transportability analysis. Hence, we expect the joint analysis to be more efficient than the other two approaches. In the Appendix, we formalize that intuition in the context of doubly robust estimators, where we show that the asymptotic variance of the estimator from the joint analysis is smaller than or equal to the asymptotic variance of both the estimators from the observational and the transportability analysis. In the Appendix, we show results from simulations comparing variance and bias of the three estimators for varying sample sizes and varying ratios of the sample size of the randomized trial and the sample size of the observational database. The results show that the variance of the joint analysis is always lower (or at least not larger) than the variance of the observational and transportability estimators.

## Application to CASS data

**Data and implementation:** We applied our methods to data from the Coronary Artery Surgery Study (CASS), a comprehensive cohort study that enrolled participants from 1975 to 1979 with end of follow-up in 1996. CASS compared the effects of coronary artery bypass grafting surgery plus medical therapy (hereafter surgery) versus only medical therapy among patients with significant coronary artery disease with a reduced ejection fraction [42, 43]. In CASS, participants could select to be a part of a randomized trial ($S=1$) and if they declined they were offered to participate in an observational study ($S=0$). As there was no censoring and previous analysis of the same data showed minimal impact of adjusting for missing data [44], we conducted a complete case analysis consisting of 1,686 participants and participant baseline characteristics stratified by study component (randomized or observational) and treatment assignment are shown in Table 1 (in this analysis $X = X^*$ and the covariates $X$ are listed in Table 1). Here, we present results from a random forest model fit on a training set comprising 50% of the source population observations, with 10-year mortality as the outcome. Using the remaining 50% of the observations in the observational study and all the randomized trial data, we calculated the Brier score for the random forest model in the population underlying the observational component for counterfactual deterministic treatment strategies of $A=1$ and $A=0$. We did that estimation using transportability, observational, and joint analysis using outcome model, inverse weighting, and doubly robust estimators. The models needed for implementation of the estimators were main effect logistic regression models.

**Table 1:**
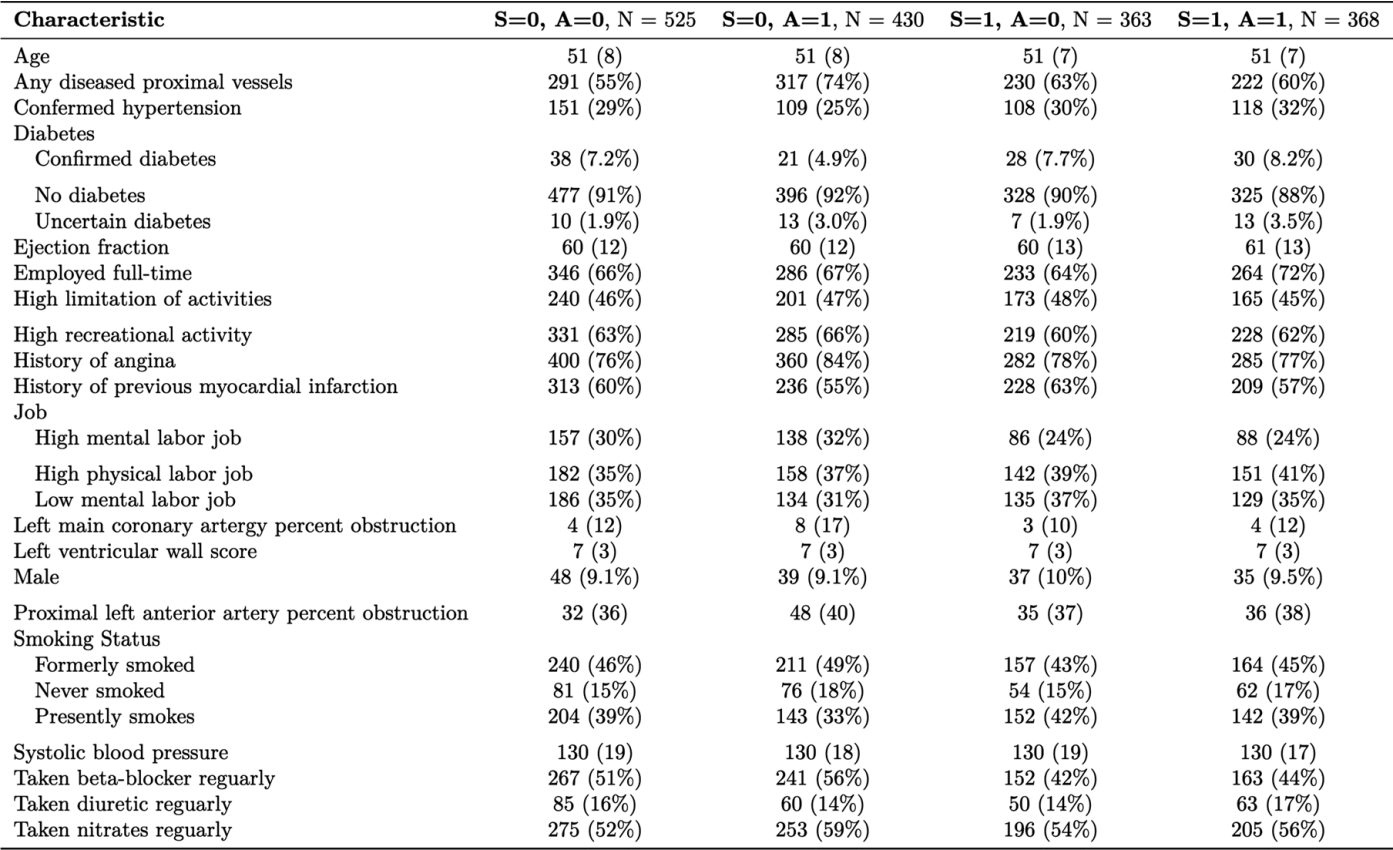
Baseline characteristics of CASS participants, stratified by study component (observational or randomized) and treatment assignment. For continuous variables we present mean (standard deviation) and for categorical we present number in each category (percent). Here, $S=1$ denotes participants who were in the randomized component of CASS, $S=0$ denotes participant in the observational component of CASS, $A=1$ denotes the surgery arm and $A=0$ denotes the medical intervention arm

**Fig. 3:**
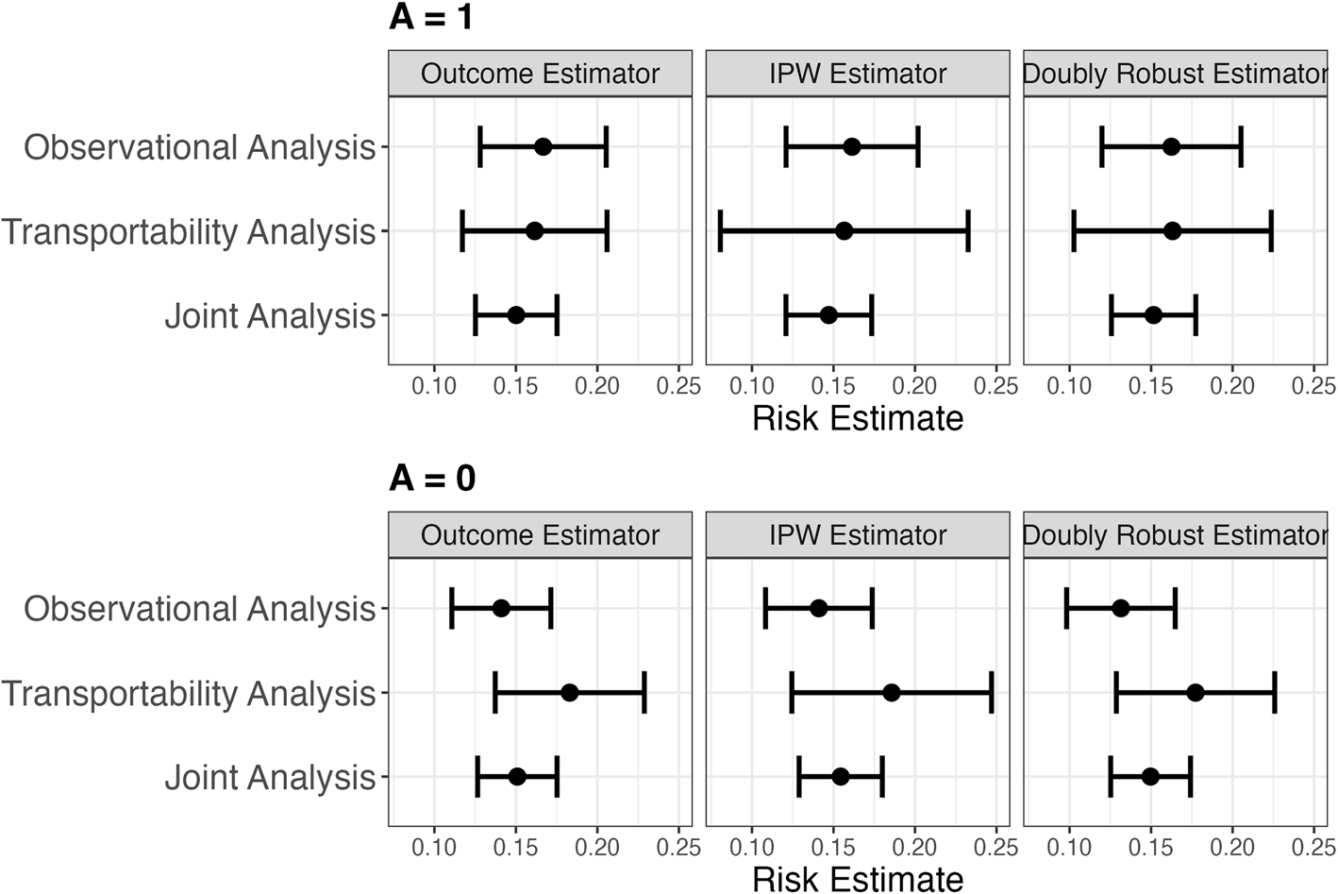
Estimates and 95% confidence intervals of Brier risk in the population underlying the observational component of CASS for counterfactual treatments $A=1$ (top) and $A=0$ (bottom). Estimates are presented for the transportability, observational, and joint analysis. For each analysis, we present estimates calculated using the outcome model, inverse-probability-weighting (IPW) and doubly-robust estimators. 95% Wald confidence intervals were obtained using the non-parametric bootstrap with 500 bootstrap samples

**Results:** Risk estimates and 95% bootstrap-based confidence intervals are shown in Fig. 3. For counterfactual treatment assignment $A=1$, all estimates are similar and the associated confidence intervals are highly overlapping. From a benchmarking perspective, this increases our confidence that the identifiability assumptions are satisfied. Furthermore, the joint analysis estimates have narrower confidence intervals than both the observational and transportability estimates. For treatment $A=0$, while there is still substantial overlap between the confidence intervals, we see a minor discordance between the observational and transportability analysis estimates, suggesting a potential mild violation of at least one of assumptions A2 or A4*. For example, prior aortic or peripheral intervention has been identified as an important predictor for long term mortality in patients with coronary artery disease [45] and that information was not collected in CASS which might lead to assumption violations. In the Appendix, we provide results from (i) the same analysis for tailored models and the trends seen are similar to those seen in Fig. 3 and (ii) results that include analysis that uses only data from the randomized component of CASS.

## Discussion

In this manuscript we discuss three ways, observational analysis, transportability analysis, and joint analysis to estimate the counterfactual risk in the target population underlying an observational study when we have data from a randomized trial and an observational study emulating the randomized trial. We also outline procedures for fitting a machine learning model that is tailored to the conditional counterfactual mean in the population underlying the observational study. We compare the assumptions needed for these approaches and provide and derive properties of estimators for joint analysis of the two datasets. One advantage of our approach is that the methods we have outlined are agnostic to the underlying structure of the machine learning model and it can also be used with traditional statistical models.

While discussed here in the context of a randomized trial and an observational study, the transportability analysis can be used more generally in situations when it is necessary to simultaneously adjust for differences in treatment strategies and covariate distributions between the two populations. For example, the transportability analysis can used when both datasets are observational studies, given that we have reason to believe *A*1* through *A*5* hold. However, in this case, one may have lower confidence in assump *A*2* (conditional exchangeability between treatment groups), since we can no longer rely on randomization in the trial.

As discussed, both the observational and transportability analysis rely on untestable assumptions and ideally subject matter knowledge should be used to determine the plausibility of each untestable assumption. However, when subject matter knowledge is insufficient to make that determination, sensitivity analysis methods that evaluate how violations of each assumption impact the findings are useful and development of such methods is of interest. There are several other interesting avenues for future research including extensions to censoring, measurement error, non-adherence to treatment assignment, and methods that do not require individual participant data. In highly related transportability analysis settings, it has been shown that correctly specified maximum likelihood estimators (MLEs) without using any data from the target population are minimax optimal in the target population (under some identifiability assumptions and when $X = X^*$) but for misspecified MLE models inverse-odds weighted MLEs improve over unweighted MLEs. It is of interest to provide similar results for the setting considered here [46, 47].

When evaluating the model, we assume that the machine learning model is built using data that is independent from the data used for evaluation of model performance. That assumption incorporates several common settings such as models that are built on an external dataset, a split into a training and a test set, and evaluating the performance of an externally developed biomarker. In our setup we assumed that the set of covariates used to adjust for confounding or between population differences $\left(X\right)$ can be larger than the set of covariates needed for the machine learning model ($X^*$). This is useful as the variables to include in the machine learning model are often selected with clinical constraints on data availability across a variety of settings in mind (e.g., avoiding covariates that are expensive or invasive to collect). Although not explicit in our notation, the set of covariates available in the observational study might be larger than the set of covariates used in the randomized trial. If that is the case, then the observational analysis can adjust for more factors than the transportability analysis as the latter is restricted to adjusting for variables that are available in both datasets.

Finally, we note that the required assumptions for any of the procedures outlined here are untestable. However, the benchmarking procedure we describe can help identify discrepancies between the observational and transportability analyses that may point to certain assumption violations, or in the case where both analyses return similar results, it can increase our confidence in the assumptions and analysis.

## Supplementary Information

Supplementary Material 1

## Data Availability

The CASS dataset can be requested from https://biolincc.nhlbi.nih.gov/studies/cass/ The code used for analysis can be found at https://github.com/sarah-voter/counterfactual prediction machine learning paper code.
