# Supplementary material for: Counterfactual prediction from machine learning models: transportability and joint analysis for model development and evaluation using multi-source data
Source: Diagn Progn Res. 2025 Oct 2;9:22. doi: 10.1186/s41512-025-00201-y (PMC12490139; doi:10.1186/s41512-025-00201-y)
Supplement: Supplementary file 1 — Supplementary Material 1 [file 41512_2025_201_MOESM1_ESM.pdf]

APPENDIX

1 Proofs of identifiability

Observational analysis: Using the assumptions made we have

$$
\begin{aligned}
&\mathrm{E}[L(Y^a, g(X^*))|S=0] & \\
&= \mathrm{E}[\mathrm{E}[L(Y^a, g(X^*))|X, S=0]|S=0] & \text{law of iterated expectation}\\
&= \mathrm{E}[\mathrm{E}[L(Y^a, g(X^*))|X, S=0, A=a]|S=0] & \textbf{Key assumption } \; Y^a \perp\!\!\!\perp \mathbf{A}|X\\
&= \mathrm{E}[\mathrm{E}[L(Y, g(X^*))|X, S=0, A=a]|S=0] & \text{consistency in } S=0\text{: if A=a, } Y^a = Y\\
&= \psi_{obs} & \text{by definition}
\end{aligned}
$$

The expectations in $\mathrm{E}[\mathrm{E}[L(Y, g(X^*))|X, S=0, A=a]|S=0]$ are well defined by the positivity assumption.

Transportability analysis:

$$
\begin{aligned}
&\mathrm{E}[L(Y^a, g(X^*))|S=0] & \\
&= \mathrm{E}[\mathrm{E}[L(Y^a, g(X^*))|X, S=0]|S=0] & \text{law of iterated expectation}\\
&= \mathrm{E}[\mathrm{E}[L(Y^a, g(X^*))|X, S=1]|S=0] & \textbf{Key assumption: } \; Y^a \perp\!\!\!\perp \mathbf{S}|X\\
&= \mathrm{E}[\mathrm{E}[L(Y^a, g(X^*))|X, S=1, A=a]|S=0] & Y^a \perp\!\!\!\perp A|X \text{ (supported by design)}\\
&= \mathrm{E}[\mathrm{E}[L(Y, g(X^*))|X, S=1, A=a]|S=0] & \text{consistency: if A=a, } Y^a = Y\\
&= \psi_{tr} & \text{by definition}
\end{aligned}
$$

Or equivalently through the inverse-weighting expression:

$$
\begin{aligned}
&\mathrm{E}[\mathrm{E}[L(Y, g(X^*))|X, S=1, A=a]|S=0]\\
&= \frac{1}{\Pr[S=0]} \mathrm{E}\left[I(S=0)\, \mathrm{E}\left[\frac{I(S=1, A=a) L(Y, g(X^*))}{\Pr[A=a, S=1|X]} \middle| X \right]\right]
\end{aligned}
$$

The expectations in $\mathrm{E}[\mathrm{E}[L(Y, g(X^*))|X, S=1, A=a]|S=0]$ are well defined by the positivity assumptions.

2 Nested Design

Nested Design

Now suppose the trial population is nested within the population underlying the observational database. In this case, our target parameter, which we will refer to as $\psi'(a)$ is the expected loss among the full population (i.e. the union of the source and target populations):

$$\psi'(a) = \mathrm{E}[L(Y^a, g(X^*))]$$

Similarly to the non-nested case, the target population can be written as the observed data functional:

$$\begin{aligned}
\mathrm{E}[L(Y^a, g(X^*))] &= \mathrm{E}[\mathrm{E}[L(Y^a, g(X^*))|X]] \\
&= \mathrm{E}[\mathrm{E}[L(Y^a, g(X^*))|X, S = 0]] \\
&= \mathrm{E}[\mathrm{E}[L(Y^a, g(X^*))|X, S = 0, A = a]] \\
&= \mathrm{E}[\mathrm{E}[L(Y, g(X^*))|X, S = 0, A = a]] \\
&= \psi'_{obs}(a)
\end{aligned}$$

And we can obtain the following identifiability using information from the source population:

$$\begin{aligned}
\mathrm{E}[L(Y^a, g(X^*))] &= \mathrm{E}[\mathrm{E}[L(Y^a, g(X^*))|X]] \\
&= \mathrm{E}[\mathrm{E}[L(Y^a, g(X^*))|X, S = 1]] \\
&= \mathrm{E}[\mathrm{E}[L(Y^a, g(X^*))|X, S = 1, A = a]] \\
&= \mathrm{E}[\mathrm{E}[L(Y, g(X^*))|X, S = 1, A = a]] \\
&= \psi'_{tr}(a)
\end{aligned}$$

Finally, we can use the information in both populations to obtain the identifiablity result for the joint analysis:

$$\begin{aligned}
\mathrm{E}[L(Y^a, g(X^*))] =& \mathrm{E}[\mathrm{E}[L(Y^a, g(X^*))|X]] \\
=& \mathrm{E}[\mathrm{E}[L(Y^a, g(X^*))|X, A = a]] \\
=& \mathrm{E}[\mathrm{E}[L(Y, g(X^*))|X, A = a]] \\
=& \psi'_{joint}(a)
\end{aligned}$$

3 Doubly robust estimators

An alternative to the estimators from the observational and transportability analysis presented in the main text is to use doubly robust estimators. For the observational analysis the doubly robust estimator [1] is given by

$$\frac{1}{n_0}\sum_{i=1}^{n} I(S_i = 0)\left(\widehat{h}_{a,0}(X_i) + \frac{I(A_i = a)}{\widehat{e}_{a,0}(X_i)}\big(L(Y_i, g(X_i^*)) - \widehat{h}_{a,0}(X_i)\big)\right).$$

where $\widehat{e}_{a,s}(X)$ is an estimator for $\Pr[A = a|X, S = s]$ for $s \in \{0, 1\}$. This estimator is consistent for the counterfactual risk in the target population if either $\widehat{e}_{a,s}(X)$ or $\widehat{h}_{a,0}(X)$ is consistent but not necessary both.

For the transportability analysis the doubly robust estimator [2] is given by

$$\frac{1}{n_0}\sum_{i=1}^{n}\left(I(S_i = 0)\widehat{h}_{a,1}(X_i) + \frac{\widehat{p}(X_i)I(S_i = 1, A_i = a)}{(1 - \widehat{p}(X_i))\widehat{e}_{a,1}(X_i)}\big(L(Y_i, g(X_i^*)) - \widehat{h}_{a,1}(X_i)\big)\right),$$

where $\widehat{p}(X)$ is an estimator for $\Pr[S = 0|X]$. The doubly robust estimator is consistent if either $\widehat{h}_{a,1}(X)$ is consistent or both $\widehat{p}(X)$ and $\widehat{e}_{a,1}(X)$ are consistent (as treatment assignment is randomized $\Pr[A = a|X, S = 1]$ is know and $\widehat{e}_{a,1}(X)$ can always be correctly specified, but estimating it can improve efficiency [3].

4 Estimators for general treatment assignment mechanisms

Now we present results for how to build a model for the conditional expectation of the potential outcome under a treatment strategy $f^*(A|X)$ in the target population and evaluate the performance of a model in the target population under a treatment strategy $f^*(A|X)$. That is we want to estimate $E_{f^*(A|X)}[Y^a|X^*, S=0]$ where X^* is a subset of X and where the subscript $f^*(A|X)$ on the expectation denotes that the expectation is taken with respect to a density that involves the counterfactual treatment strategy $f^*(A|X)$ (as well as other densities).

The identifiability result for the observational analysis says that for a general treatment assignment strategy $f^*(A|X)$ the counterfactual risk in the target population can, under assumptions A1 through A3, be written as

$$E_{f^*(A|X)}[\mathrm{E}[L(Y, g(X^*))|X, S=0, A=a]|S=0].$$

and the corresponding sample analog is given by

$$\widehat{\psi}_{obs} = \frac{1}{n_0}\sum_{i=1}^{n} I(S_i=0)\left(\Pr[A=1|X_i]\widehat{h}_{1,0}(X_i) + \Pr[A=0|X_i]\widehat{h}_{0,0}(X_i)\right)$$

where $\Pr[A=a|X]$ is the probability of receiving treatment $a \in \{0,1\}$ under the treatment assignment strategy $f^*(A|X)$. That is, $\Pr[A=a|X]$ is user defined and not specified by the data (e.g., if interest lies in evaluating counterfactual performance if everyone is assigned treatment $A=1$, then $\Pr[A=1|X=x]=1$ and $\Pr[A=0|X=x]=0$ for all x).

For the transportability analysis the counterfactual risk in the target population can, under assumptions A1* through A5*, be written as

$$E_{f^*(A|X)}[\mathrm{E}[L(Y, g(X^*))|X, S=1, A=a]|S=0].$$

The sample analog of this identifiability result gives the transportability estimator for a general

treatment strategy

$$\widehat{\psi}_{tr} = \frac{1}{n_0}\sum_{i=1}^{n} I(S_i = 0)\Big(\Pr[A = 1|X_i]\widehat{h}_{1,1}(X_i) + \Pr[A = 0|X_i]\widehat{h}_{0,1}(X_i)\Big)$$

5 Additional details on joint analysis

If assumptions A1 through A3 and A1* through A5* hold, then the counterfactual risk in the target population can be written as

$$\begin{aligned}
\mathrm{E}[L(Y^a, g(X^*))|S = 0] &= \mathrm{E}[\mathrm{E}[L(Y^a, g(X^*))|X, S = 0]|S = 0] \\
&= \mathrm{E}[\mathrm{E}[L(Y^a, g(X^*))|X]|S = 0] \\
&= \mathrm{E}[\mathrm{E}[L(Y^a, g(X^*))|X, A = a]|S = 0] \\
&= \mathrm{E}[\mathrm{E}[L(Y, g(X^*))|X, A = a]|S = 0]
\end{aligned}$$

Or equivalently through the inverse-weighting expression

$$\begin{aligned}
&\mathrm{E}[\mathrm{E}[L(Y, g(X^*))|X, A = a]|S = 0] \\
&= \frac{1}{\Pr[S = 0]}\,\mathrm{E}\left[I(S = 0)\,\mathrm{E}\left[\frac{I(A = a)L(Y, g(X^*))}{\Pr[A = a|X]}\middle| X\right]\right] \\
&= \frac{1}{\Pr[S = 0]}\,\mathrm{E}\left[\mathrm{E}\left[\frac{\Pr[S = 0|X]I(A = a)L(Y, g(X^*))}{\Pr[A = a|X]}\middle| X\right]\right] \\
&= \frac{1}{\Pr[S = 0]}\,\mathrm{E}\left[\frac{\Pr[S = 0|X]I(A = a)L(Y, g(X^*))}{\Pr[A = a|X]}\right].
\end{aligned}$$

The inverse-weighting expression suggests the inverse-weighting estimator

$$\widehat{\psi}_{joint,iw} = \frac{1}{n_0}\sum_{i=1}^{n}\frac{\widehat{p}(X_i)I(A_i = a)L(Y_i, g(X_i^*))}{\widehat{e}_a(X_i)},$$

where $\widehat{e}_a(X)$ is an estimator for $\Pr[A = a|X]$ and $\widehat{p}(X)$ is an estimator $\Pr[S = 0|X]$.

An alternative estimator is a "doubly robust" estimator

$$\widehat{\psi}_{joint,dr} = \frac{1}{n_0}\sum_{i=1}^{n}\left(\frac{\widehat{p}(X_i)I(A_i = a)}{\widehat{e}_a(X_i)}\left(L(Y_i, g(X_i^*)) - \widehat{h}_a(X_i)\right) + I(S_i = 0)\widehat{h}_a(X_i)\right).$$

We refer to this as the doubly robust estimator as it has the following property

Theorem 1. *The doubly robust estimator* $\widehat{\psi}_{joint,dr}$ *is consistent if either* $\widehat{h}_a(X)$ *converges to* $\mathrm{E}[L(Y, g(X^*))|X, A = a]$ *or* $\widehat{p}(X)$ *converges to* $\Pr[S = 0|X]$ *and* $\widehat{e}_a(X)$ *converges to* $\Pr[A = a|X]$.

Proof: Let $p^*(X)$ be the asymptotic limit of $\widehat{p}(X)$, $e_a^*(X)$ be the asymptotic limit of $\widehat{e}_a(X)$, and $h_a^*(X)$ be the asymptotic limit of $\widehat{h}(X)$. The doubly robust estimator converges to

$$\frac{1}{\Pr[S = 0]}\,\mathrm{E}\left[\frac{p^*(X)I(A = a)}{e_a^*(X)}\left(L(Y, g(X^*)) - h_a^*(X)\right) + I(S = 0)h_a^*(X)\right]$$

Case 1: First assume that $p^*(X) = \Pr[S = 0|X]$ and $e_a^*(X) = \Pr[A = a|X]$, but we make no assumptions on $h_a^*(X)$. Then, the doubly robust estimator converges to

$$\frac{1}{\Pr[S = 0]}\,\mathrm{E}\left[\frac{\Pr[S = 0|X]I(A = a)}{\Pr[A = a|X]}\left(L(Y, g(X^*)) - h_a^*(X)\right) + I(S = 0)h_a^*(X)\right]$$

Now

$$\begin{aligned}
&\mathrm{E}\left[\frac{\Pr[S = 0|X]I(A = a)}{\Pr[A = a|X]}L(Y, g(X^*))\right]\\
&= \mathrm{E}\left[\mathrm{E}\left[\frac{\Pr[S = 0|X]I(A = a)}{\Pr[A = a|X]}L(Y, g(X^*))|X\right]\right]\\
&= \mathrm{E}\left[I(S = 0)\,\mathrm{E}\left[\frac{I(A = a)}{\Pr[A = a|X]}L(Y, g(X^*))|X\right]\right]\\
&= \mathrm{E}\left[I(S = 0)\,\mathrm{E}\left[L(Y, g(X^*))|X, A = a\right]\right]
\end{aligned}$$

So,

$$\frac{1}{\Pr[S = 0]}\,\mathrm{E}\left[\frac{\Pr[S = 0|X]I(A = a)}{\Pr[A = a|X]}L(Y, g(X^*))\right] = \mathrm{E}\left[\mathrm{E}\left[L(Y, g(X^*))|X, A = a\right]|S = 0\right]$$

We have

$$
\begin{aligned}
&\mathrm{E}\left[\frac{\Pr[S=0|X]I(A=a)}{\Pr[A=a|X]}h_a^*(X)\right] \\
&\mathrm{E}\left[\mathrm{E}\left[\frac{\Pr[S=0|X]I(A=a)}{\Pr[A=a|X]}h_a^*(X)\middle|X\right]\right] \\
&=\mathrm{E}\left[I(S=0)\,\mathrm{E}\left[h_a^*(X)|X,A=a\right]\right] \\
&=\mathrm{E}\left[I(S=0)h_a^*(X)\right]
\end{aligned}
$$

completing the proof of the first case.

Case 2: Now assume that $h_a^*(X) = \mathrm{E}[L(Y, g(X^*))|X, A = a]$, but we make no assumptions on the form of $p^*(X)$ and $e_a^*(X)$. Then, the doubly robust estimator converges to

$$
\frac{1}{\Pr[S=0]}\,\mathrm{E}\left[\frac{p^*(X)I(A=a)}{e^*(X)}\left(L(Y,g(X^*))-\mathrm{E}[L(Y,g(X^*))|X,A=a]\right)+I(S=0)\,\mathrm{E}[L(Y,g(X^*))|X,A=a]\right].
$$

The result follows as

$$
\frac{1}{\Pr[S=0]}\,\mathrm{E}\left[I(S=0)\,\mathrm{E}[L(Y,g(X^*))|X,A=a]\right]=\mathrm{E}\left[\mathrm{E}[L(Y,g(X^*))|X,A=a]|S=0\right]
$$

and

$$
\begin{aligned}
&\mathrm{E}\left[\frac{p^*(X)I(A=a)}{e^*(X)}\left(L(Y,g(X^*))-\mathrm{E}\left[L(Y,g(X^*))|X,A=a\right]\right)\right] \\
&=\mathrm{E}\left[\mathrm{E}\left[\frac{p^*(X)I(A=a)}{e^*(X)}\left(L(Y,g(X^*))-\mathrm{E}\left[L(Y,g(X^*))|X,A=a\right]\right)\middle|X\right]\right] \\
&=\mathrm{E}\left[\mathrm{E}\left[\frac{p^*(X)\Pr[A=a|X]}{e^*(X)}\left(L(Y,g(X^*))-\mathrm{E}\left[L(Y,g(X^*))|X,A=a\right]\right)\middle|X,A=a\right]\right] \\
&=0
\end{aligned}
$$

$\square$

Comparisons of variance of double robust estimators

If all identifiability assumptions hold (A1 through A3 and A1* through A5*) we presented three ways of estimating the counterfactual risk in the target population, the observational analysis, transportability analysis, and the joint analysis. Now we show that if a doubly robust estimator is used, then the joint analysis has smaller asymptotic variance than both the observational and the transportability analysis.

The influence function for the observational analysis is given by

$$IF_{obs}(a) = \frac{1}{\Pr[S=0]} \operatorname{E}\Bigg[\frac{I(S=0, A=a)}{\Pr[A=a|X, S=0]} \left(L(Y, g(X^*)) - \operatorname{E}[L(Y, g(X^*))|X, S=0, A=a]\right) \\ + I(S=0) \left(E[L(Y, g(X^*))|X, S=0, A=a] - \psi_{obs}(a)\right) \Bigg]$$

The influence function for the transportability analysis is given by

$$IF_{tr}(a) = \frac{1}{\Pr[S=0]} \operatorname{E}\Bigg[\frac{I(S=1, A=a) \Pr[S=0|X]}{\Pr[A=a|X, S=1] \Pr[S=1|X]} \left(L(Y, g(X^*)) - \operatorname{E}[L(Y, g(X^*))|X, S=1, A=a]\right) \\ + I(S=0) \left(E[L(Y, g(X^*))|X, S=1, A=a] - \psi_{tr}(a)\right) \Bigg]$$

The influence function for the joint analysis is given by

$$IF_{joint}(a) = \frac{1}{\Pr[S=0]} \operatorname{E}\Bigg[\frac{I(A=a) \Pr[S=0|X]}{\Pr[A=a|X]} \left(L(Y, g(X^*)) - \operatorname{E}[L(Y, g(X^*))|X, A=a]\right) \\ + I(S=0) \left(E[L(Y, g(X^*))|X, A=a] - \psi_{joint}(a)\right) \Bigg]$$

For each analysis, the asymptotic variance of the doubly robust estimator is given by the square of the influence function.

We can rewrite the asymptotic variance for the observational analysis estimator as

$$\mathrm{E}[(IF_{obs}(a))^2] = \frac{1}{\Pr[S=0]^2}\,\mathrm{E}\Bigg[\left(\frac{I(S=0,A=a)}{\Pr[A=a|X,S=0]}\left(L(Y,g(X^*)) - \mathrm{E}[L(Y,g(X^*))|X,S=0,A=a]\right)\right)^2 \\ + \left(I(S=0)\left(E[L(Y,g(X^*))|X,S=0,A=a] - \psi_{obs}(a)\right)\right)^2\Bigg],$$

the transportability estimator as

$$\mathrm{E}[(IF_{tr}(a))^2] = \\ \frac{1}{\Pr[S=0]^2}\,\mathrm{E}\Bigg[\left(\frac{I(S=1,A=a)\Pr[S=0|X]}{\Pr[A=a|X,S=1]\Pr[S=1|X]}\left(L(Y,g(X^*)) - \mathrm{E}[L(Y,g(X^*))|X,S=1,A=a]\right)\right)^2 \\ + \left(I(S=0)\left(E[L(Y,g(X^*))|X,S=1,A=a] - \psi_{tr}(a)\right)\right)^2\Bigg]$$

and for the joint analysis estimator

$$\mathrm{E}[(IF_{joint}(a))^2] = \frac{1}{\Pr[S=0]^2}\,\mathrm{E}\Bigg[\left(\frac{I(A=a)\Pr[S=0|X]}{\Pr[A=a|X]}\left(L(Y,g(X^*)) - \mathrm{E}[L(Y,g(X^*))|X,A=a]\right)\right)^2 \\ + \left(I(S=0)\left(E[L(Y,g(X^*))|X,A=a] - \psi_{joint}(a)\right)\right)^2\Bigg]$$

By the assumptions made

$$\psi_{joint}(a) = \psi_{tr}(a) = \psi_{obs}(a),$$

and

$$E[L(Y,g(X^*))|X,A=a] = E[L(Y,g(X^*))|X,S=1,A=a] = E[L(Y,g(X^*))|X,S=0,A=a].$$

This implies that

$$\mathrm{E}\left[\left(I(S=0)(E[L(Y,g(X^*))|X,A=a] - \psi_{joint}(a))\right)^2\right] \\ = \mathrm{E}\left[\left(I(S=0)(E[L(Y,g(X^*))|X,S=1,A=a] - \psi_{tr}(a)\right)^2\right] \\ = \mathrm{E}\left[\left(I(S=0)(E[L(Y,g(X^*))|X,S=0,A=a] - \psi_{obs}(a)\right)^2\right].$$

So it is enough to compare the first term inside the expectation of the square of each influence function. For the estimator from the observational analysis that term is given by

$$
\begin{aligned}
&\mathrm{E}\left[\left(\frac{I(S=0,A=a)}{\Pr[A=a|X,S=0]}\left(L(Y,g(X^*)) - \mathrm{E}[L(Y,g(X^*))|X,S=0,A=a]\right)\right)^2\right] \\
&= \mathrm{E}\left[\mathrm{E}\left[\frac{I(S=0,A=a)}{\Pr[A=a|X,S=0]^2}\left(L(Y,g(X^*)) - \mathrm{E}[L(Y,g(X^*))|X,S=0,A=a]\right)^2 \middle| X\right]\right] \\
&= \mathrm{E}\left[\mathrm{E}\left[\frac{\Pr[S=0|X]}{\Pr[A=a|X,S=0]}\left(L(Y,g(X^*)) - \mathrm{E}[L(Y,g(X^*))|X,S=0,A=a]\right)^2 \middle| X,S=0,A=a\right]\right] \\
&= \mathrm{E}\left[\frac{\Pr[S=0|X]}{\Pr[A=a|X,S=0]} Var[L(Y,g(X^*))|X,S=0,A=a]\right]
\end{aligned}
$$

For the estimator from the joint analysis that term is given by

$$
\begin{aligned}
&\mathrm{E}\left[\left(\frac{I(A=a)\Pr[S=0|X]}{\Pr[A=a|X]}\left(L(Y,g(X^*)) - \mathrm{E}[L(Y,g(X^*))|X,A=a]\right)\right)^2\right] \\
&\mathrm{E}\left[\mathrm{E}\left[\frac{I(A=a)\Pr[S=0|X]^2}{\Pr[A=a|X]^2}\left(L(Y,g(X^*)) - \mathrm{E}[L(Y,g(X^*))|X,A=a]\right)^2 \middle| X\right]\right] \\
&\mathrm{E}\left[\mathrm{E}\left[\frac{\Pr[S=0|X]^2}{\Pr[A=a|X]}\left(L(Y,g(X^*)) - \mathrm{E}[L(Y,g(X^*))|X,A=a]\right)^2 \middle| X,A=a\right]\right] \\
&\mathrm{E}\left[\frac{\Pr[S=0|X]^2}{\Pr[A=a|X]} Var[L(Y,g(X^*))|X,A=a]\right]
\end{aligned}
$$

The identifiability assumptions guarantee that

$$
Var[L(Y,g(X^*))|X,A=a] = Var[L(Y,g(X^*))|X,S=1,A=a] = Var[L(Y,g(X^*))|X,S=0,A=a]. \tag{1}
$$

and

$$
\Pr[A=a|X,S=1]\Pr[S=1|X] > 0.
$$

And standard calculations give

$$
\Pr[A=a|X] = \Pr[A=a|X,S=1]\Pr[S=1|X] + \Pr[A=a|X,S=0]\Pr[S=0|X] \tag{2}
$$

Combining this gives

$$
\begin{aligned}
&\mathrm{E}\left[\frac{\Pr[S=0|X]^2}{\Pr[A=a|X]} Var[L(Y,g(X^*))|X,A=a]\right] \\
&= \mathrm{E}\left[\frac{\Pr[S=0|X]^2}{\Pr[A=a|X,S=1]\Pr[S=1|X]+\Pr[A=a|X,S=0]\Pr[S=0|X]} Var[L(Y,g(X^*))|X,S=0,A=a]\right] \\
&< \mathrm{E}\left[\frac{\Pr[S=0|X]^2}{\Pr[A=a|X,S=0]\Pr[S=0|X]} Var[L(Y,g(X^*))|X,S=0,A=a]\right] \\
&= \mathrm{E}\left[\frac{\Pr[S=0|X]}{\Pr[A=a|X,S=0]} Var[L(Y,g(X^*))|X,S=0,A=a]\right].
\end{aligned}
$$

This shows that the asymptotic variance of the estimator from the joint analysis is less than the asymptotic variance from the observational analysis.

For the transportability estimator similar calculations give

$$
\begin{aligned}
&\mathrm{E}\left[\left(\frac{I(S=1,A=a)\Pr[S=0|X]}{\Pr[A=a|X,S=1]\Pr[S=1|X]}\left(L(Y,g(X^*)) - \mathrm{E}[L(Y,g(X^*))|X,S=1,A=a]\right)\right)^2\right] \\
&= \mathrm{E}\left[\mathrm{E}\left[\frac{I(S=1,A=a)\Pr[S=0|X]^2}{\Pr[A=a|X,S=1]^2\Pr[S=1|X]^2}\left(L(Y,g(X^*)) - \mathrm{E}[L(Y,g(X^*))|X,S=1,A=a]\right)^2 \middle| X\right]\right] \\
&= \mathrm{E}\left[\frac{\Pr[S=0|X]^2}{\Pr[A=a|X,S=1]\Pr[S=1|X]} Var[L(Y,g(X^*))|X,S=1,A=a]\right].
\end{aligned}
$$

So, using equations (1) and (2)

$$
\begin{aligned}
&\mathrm{E}\left[\frac{\Pr[S=0|X]^2}{\Pr[A=a|X]} Var[L(Y,g(X^*))|X,A=a]\right] \\
&= \mathrm{E}\left[\frac{\Pr[S=0|X]^2}{\Pr[A=a|X,S=1]\Pr[S=1|X]+\Pr[A=a|X,S=0]\Pr[S=0|X]} Var[L(Y,g(X^*))|X,S=1,A=a]\right] \\
&\leq \mathrm{E}\left[\frac{\Pr[S=0|X]^2}{\Pr[A=a|X,S=1]\Pr[S=1|X]} Var[L(Y,g(X^*))|X,S=1,A=a]\right]
\end{aligned}
$$

This shows that the asymptotic variance of the estimator from the joint analysis is less than or equal to the asymptotic variance from the transportability analysis.

6 Tailoring the Counterfactual Prediction Model

6.1 Transportability Analysis

$$
\begin{aligned}
\mathrm{E}[Y^a|X^*, S=0] &= \mathrm{E}[\mathrm{E}[Y^a|X, S=0]|X^*, S=0] && \text{by the law of iterated expectation} \\
&= \mathrm{E}[\mathrm{E}[Y^a|X, S=1]|X^*, S=0] && \text{by conditional exchangeability assumption A4*} \\
&= \mathrm{E}[\mathrm{E}[Y^a|X, S=1, A=a]|X^*, S=0] && \text{by conditional exchangeability assumption A2*} \\
&= \mathrm{E}[\mathrm{E}[Y|X, S=1, A=a]|X^*, S=0] && \text{by consistency assumption A1*}
\end{aligned}
$$

Or we can obtain the equivalent inverse probability weighting representation:

$$
\begin{aligned}
\mathrm{E}[Y^a|X^*, S=0] &= \mathrm{E}[\mathrm{E}[Y|X, S=1, A=a]|X^*, S=0] && \text{(as shown above)} \\
&= \mathrm{E}\left[\frac{I(S=0)}{\Pr[S=0]}\,\mathrm{E}[Y|X, S=1, A=a]|X^*\right] && \text{condition on } S=0 \\
&= \frac{1}{\Pr[S=0]}\,\mathrm{E}[\mathrm{E}[I(S=0)\,\mathrm{E}[Y|X, S=1, A=a]|X]|X^*] && \text{iterated expectations} \\
&= \frac{1}{\Pr[S=0]}\,\mathrm{E}[\mathrm{E}[I(S=0)|X]\,\mathrm{E}[Y|X, S=1, A=a]|X^*] \\
&= \frac{1}{\Pr[S=0]}\,\mathrm{E}[\Pr[S=0|X]\,\mathrm{E}[Y|X, S=1, A=a]|X^*] \\
&= \frac{1}{Pr[S=0]}\,\mathrm{E}[\mathrm{E}[Pr[S=0|X]Y|X, S=1, A=a]|X^*]
\end{aligned}
$$

$$
\begin{aligned}
&= \frac{1}{Pr[S=0]}\,\mathrm{E}\left[\mathrm{E}\left[\frac{Pr[S=0|X]I(S=1, A=a)}{Pr[S=1, A=a|X]}Y|X\right]|X^*\right] \\
&= \frac{1}{Pr[S=0]}\,\mathrm{E}\left[\frac{Pr[S=0|X]I(S=1, A=a)}{Pr[S=1, A=a|X]}Y|X^*\right] && \text{iterated expectations} \\
&= \frac{1}{Pr[S=0]}\,\mathrm{E}\left[\frac{Pr[S=0|X]I(S=1, A=a)}{Pr[A=a|X, S=1]P[S=1|X]}Y|X^*\right]
\end{aligned}
$$

6.2 Observational Analysis

As shown in [1], we can also identify $\mathrm{E}[Y^a|X^*, S = 0]$ using only the data in the observational study.

$$
\begin{aligned}
\mathrm{E}[Y^a|X^*, S=0] &= \mathrm{E}[\mathrm{E}[Y^a|X, S=0]|X^*, S=0] && \text{by the law of iterated expectation}\\
&= \mathrm{E}[\mathrm{E}[Y^a|X, S=0, A=a]|X^*, S=0] && \text{by conditional exchangeability assumption A2}\\
&= \mathrm{E}[\mathrm{E}[Y|X, S=0, A=a]|X^*, S=0] && \text{by consistency assumption A1}
\end{aligned}
$$

Again, we can use the law of iterated expectations to obtain the equivalent inverse probability weighting representation:

$$
\begin{aligned}
\mathrm{E}[Y^a|X^*, S=0] &= \mathrm{E}[\mathrm{E}[Y|X, S=0, A=a]|X^*, S=0] && \text{(as shown above)}\\
&= \mathrm{E}\left[\mathrm{E}\left[\frac{I(A=a)}{Pr[A=a|X, S=0]}Y|X, S=0\right]|X^*, S=0\right]\\
&= \mathrm{E}\left[\frac{I(A=a)}{Pr[A=a|X, S=0]}Y|X^*, S=0\right]
\end{aligned}
$$

The sample analog of the inverse probability weighting representation gives the inverse weighting estimator that is obtained by fitting a weighted model for conditional expectation of the outcome Y given X^* among the observations in the observational database with weights equal to $\frac{I(A=a)}{\widehat{\Pr}[A=a|X,S=0]}$ where $\widehat{Pr}[A = a|X, S = 0]$ is some estimator for $\Pr[A = a|X, S = 0]$.

6.3 Joint Analysis

Finally, we can identify $\text{E}[Y^a|X^*, S = 0]$ using the combined data from both the randomized trial and the observational study:

$$\begin{aligned}
\text{E}[Y^a|X^*, S = 0] &= \text{E}[\text{E}[Y^a|X, S = 0]|X^*, S = 0] \\
&= \text{E}[\text{E}[Y^a|X]|X^*, S = 0] \\
&= \text{E}[\text{E}[Y^a|X, A = a]|X^*, S = 0] \\
&= \text{E}[\text{E}[Y|X, A = a]|X^*, S = 0]
\end{aligned}$$

Inverse probability weighting representation:

$$\begin{aligned}
\text{E}[Y^a|X^*, S = 0] &= \text{E}[\text{E}[Y|X, A = a]|X^*, S = 0] \qquad \text{(as shown above)} \\
&= \frac{1}{Pr[S = 0]} \text{E}[I(S = 0)\, \text{E}[Y|X, A = a]|X^*] \\
&= \frac{1}{Pr[S = 0]} \text{E}\left[Pr[S = 0|X]\, \text{E}[Y|X, A = a] \middle| X^* \right] \\
&= \frac{1}{Pr[S = 0]} \text{E}\left[Pr[S = 0|X]\, \text{E}\left[\frac{I(A = a)}{Pr[A = a|X]} Y \middle| X \right] \middle| X^* \right] \\
&= \frac{1}{Pr[S = 0]} \text{E}\left[\frac{I(A = a) Pr[S = 0|X]}{Pr[A = a|X]} Y \middle| X^* \right] \qquad \text{iterated expectations}
\end{aligned}$$

The inverse probability weighting representation suggests fitting a weighted model for Y given X^* in the observational database with weights equal to $\frac{I(A=a)\widehat{Pr}[S=0|X]}{\widehat{Pr}[A=a|X]}$, where $\widehat{Pr}[S = 0|X]$ is a model for $Pr[S = 0|X]$ and $\widehat{Pr}[A = a|X]$ is a model for $Pr[A = a|X]$.

Additional details on the illustrative example

The table below shows details on how the data was simulated for the illustrative example presented in the main text. Each of the simulated datasets were generated to have 2,000 observations total (source and target population combined). For each case, the results are averaged over 500

simulations, with the bias calculated relative to the true risk, denoted ψ_{true}, which was calculated numerically using a large data set (10^5 points). Note that for both estimators $\widehat{\psi}_{obs}$ and $\widehat{\psi}_{tr}$, the relative bias shown in Figure 2 was calculated as follows: $relative\ bias = (\frac{1}{500}\sum_{i=1}^{500}\widehat{\psi}_i - \psi_{true})/\psi_{true}$.

$X \sim Unif(0,1)$	Covariate X is uniformly distributed between 0 and 1
$U \sim Unif(0,1)$	Unmeasured confounder U is uniformly distributed between 0 and 1
$S \sim Bernoulli\left(p = logit^{-1}(-0.5 + X)\right)$	Probability of being in the randomized trial depends on X, leading to a difference in covariate distributions between the source and target populations; centered so that the marginal probability is 0.5, giving equally sized trial and target populations
$A \sim Bernoulli(p = logit^{-1}((1-S)(-1+2X$ $+\frac{\beta_{AU}}{2} - \beta_{AU} * U)))$	Treatment assignment depends on X and U in the target population only, and the level of unmeasured confounding is modulated by β_{AU}. In the randomized trial, treatment assignment is independent of X and U to reflect marginal randomization.
$Y = 4X + 2A + \mu_{YU} * U + \varepsilon_Y$	Conditional expectation of the outcome depends on X, A, and U and the dependence of U is modulated by μ_{YU}
$Var(Y\|X,A,U) = \varepsilon_Y \sim N(\mu = 0, ...$ $sd = 2X +\ (1-S)(\sigma_{YU} * U * A)\ )$	Variance of Y is dependent on X (making X a prediction error modifier) in both populations. U only acts as a prediction error modifier in the treatment group of the *target* population, with effect size modulated by σ_{YU}
g(X) = 4X + 2A	Prediction model is correctly specified with respect to covariate X and treatment A.

Table 1: Simulation setup for the illustrative example

Figure 1 shows the relationship between Y^1 and the unmeasured covariate U for the four cases presented in the main manuscript.

Simulations: Estimator Variance Comparison

We use simulation to compare the performance of the three estimators (transportability, observational and joint analyses) when all required identifiability assumptions (A1-A3 & A1*-A5*) are satisfied. In this example, we consider six covariates drawn from a multivariate normal with $\underset{n\times 6}{X} \sim MVN_6(\boldsymbol{\mu} = \mathbf{0}, \boldsymbol{\Sigma} = (0.25)^{\boldsymbol{M}})$, where $\boldsymbol{M}_{i,j} = |i-j|$. For each simulated participant,

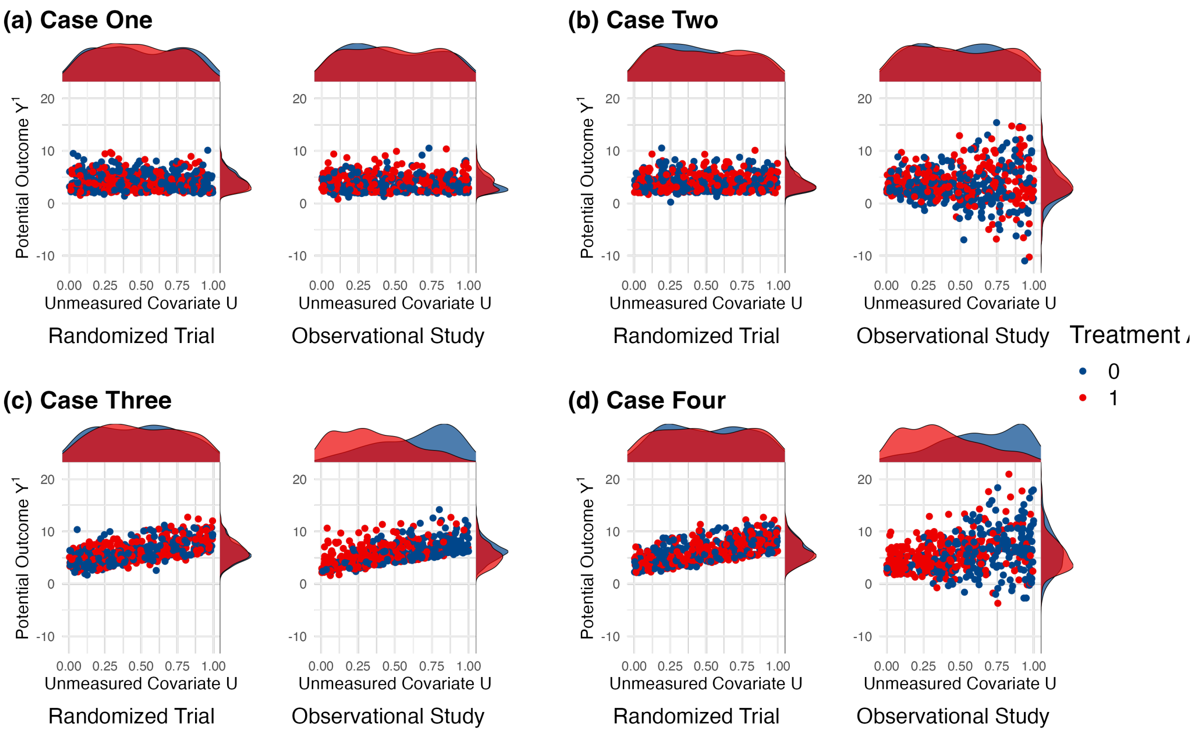

Figure 1: Visual examples of data representing each of the four cases described in this illustration. Each plot shows scatterplots of the counterfactual outcome Y^1 vs. the unobserved covariate U. The density of Y^1 by treatment group is shown on the right of each plot and the density of U by treatment group is shown above each plot.

probability of coming from the randomized trial depends on the covariates through $\Pr[S = 1|\mathbf{X}] = logit^{-1}(\boldsymbol{\beta}_{S0} + [\mathbf{X}; \mathbf{X}^2]\boldsymbol{\beta}_S)$. For participants in the randomized trial, : $\Pr[A = 1|X, S = 1] = 0.5$ for all participants reflecting marginal randomization, and in the target population (i.e. the observational database), $\Pr[A = 1|X, S = 0] = logit^{-1}(\boldsymbol{\beta}_{A0} + [\mathbf{X}; \mathbf{X}^2]\boldsymbol{\beta}_A)$. Finally, we simulated outcomes using linear model: $Y = 0.25A + \boldsymbol{\beta}_{Y0} + [\mathbf{X}; \mathbf{X}^2]\boldsymbol{\beta}_Y + \boldsymbol{\varepsilon}$, where $\boldsymbol{\varepsilon} \sim N(\mu = 0, \sigma = \boldsymbol{\beta}_{\sigma Y0} + [\mathbf{X}; \mathbf{X}^2]^2\boldsymbol{\beta}_{\sigma Y})$. Values of the coefficients $\boldsymbol{\beta}_{S0}, \boldsymbol{\beta}_S, \boldsymbol{\beta}_{A0}, \boldsymbol{\beta}_A, \boldsymbol{\beta}_{Y0}, \boldsymbol{\beta}_Y, \boldsymbol{\beta}_{\sigma Y0}, \boldsymbol{\beta}_{\sigma Y}$ can be found in the table below.. We considered varying sample sizes of $n = 500, 1000$ and 5000. In each case, the fraction of participants in the target population $\Pr[S = 0]$ was varied between 0.2 and 0.8 by changing the value of the intercept $\boldsymbol{\beta}_{S0}$ [4]. For each scenario, we conducted 1,000 simulations and the results are presented in Figure 2. For each simulation within each scenario, the scaled bias was calculated relative to the true counterfactual risk, which was estimated numerically using a large simulated dataset of size $300,000$.

As can be seen in Figure 2, across all scenarios, the joint analysis estimator demonstrated

equal or better performance (lower variance and lower bias) compared to the observational and transportability analyses. The variance of the observational and transportability estimators relative to the joint estimator was proportional to the fraction of participants in the observational database and randomized trial, respectively. As expected, the variance across all three estimators decreased as n (the size of the overall combined population) increased. In all cases, the $\frac{Bias^2}{Variance}$ ratio did not exceed a value of 0.2, suggesting that unintentional positivity assumption violations at the edge cases (smallest sample sizes or extreme source/target population distribution ratios) remained minor.

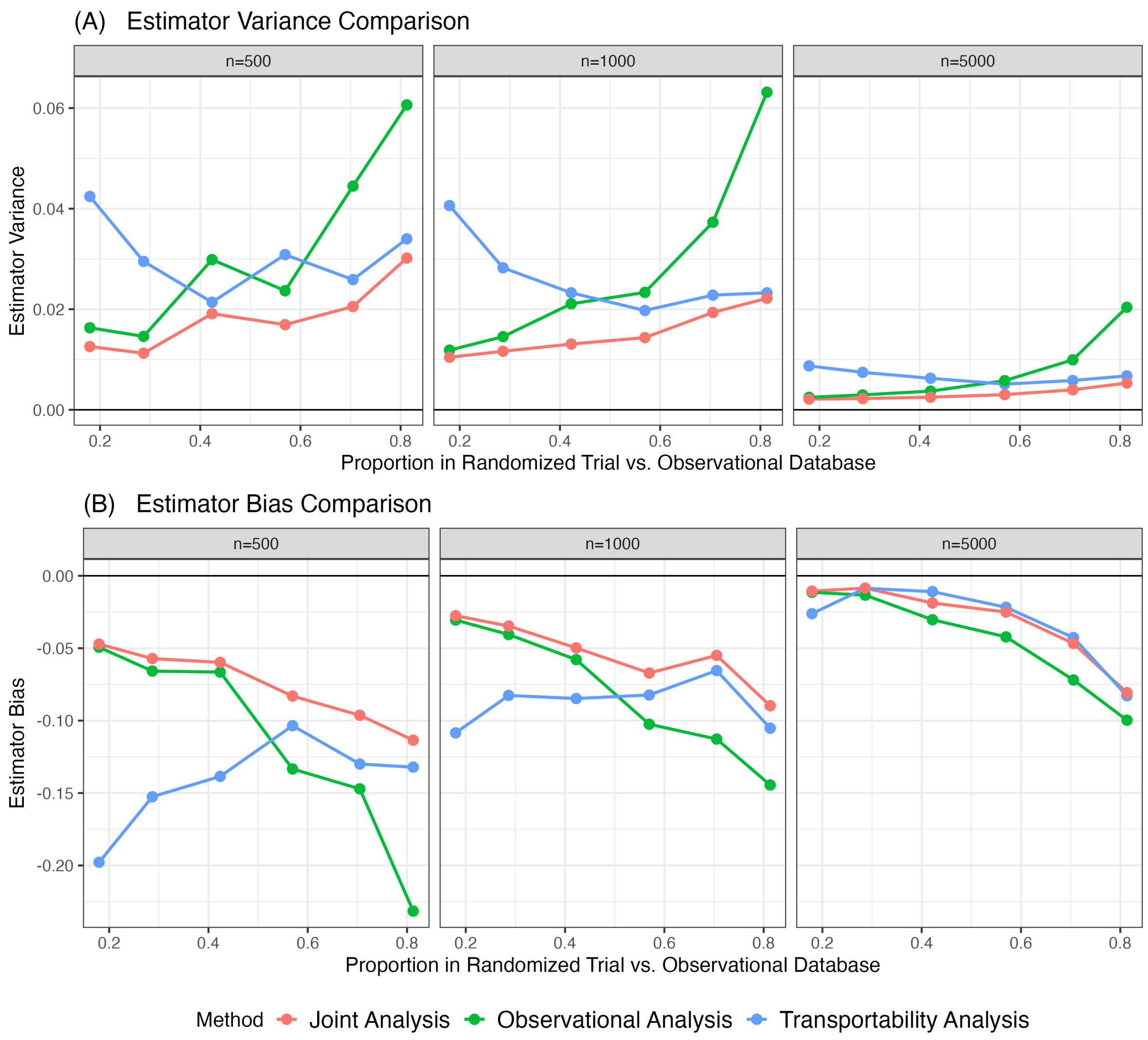

Figure 2: Simulation results showing bias and variance when the proportion of participants that are from the randomized trial varies between 0.2 and 0.8 and for sample sizes of $n = 500, 1000$ and $5,000$.

	β_S	β_A	β_Y	$\beta_{\sigma Y}$
β_{x0}	(-1.75 to 1.4)	0.00	-0.30	0.0100
β_{x1}	0.20	0.20	0.20	0.0500
β_{x2}	0.20	0.20	0.20	0.0500
β_{x3}	0.20	0.20	0.20	0.0500
β_{x4}	0.20	0.20	0.20	0.0500
β_{x5}	0.00	0.00	0.00	0.0000
β_{x6}	0.00	0.00	0.00	0.0000
β_{x7}	0.05	0.05	0.05	0.0125
β_{x8}	0.05	0.05	0.05	0.0125
β_{x9}	0.05	0.05	0.05	0.0125
β_{x10}	0.05	0.05	0.05	0.0125
β_{x11}	0.00	0.00	0.00	0.0000
β_{x12}	0.00	0.00	0.00	0.0000

Table 2: Values of $\boldsymbol{\beta}_{S0}, \boldsymbol{\beta}_S, \boldsymbol{\beta}_{A0}, \boldsymbol{\beta}_A, \boldsymbol{\beta}_{Y0}, \boldsymbol{\beta}_Y, \boldsymbol{\beta}_{\sigma Y0}, \boldsymbol{\beta}_{\sigma Y}$ used in the estimator variance simulations

References

[1] C. Boyer, *New approaches to factual and counterfactual prediction modeling.* Phd thesis, Harvard University, 2023.

[2] S. Morrison, C. Gatsonis, I. J. Dahabreh, and B. Li, "Robust Estimation of Loss-Based Measures of Model Performance under Covariate Shift," 2022.

[3] J. A. Steingrimsson, D. F. Hanley, and M. Rosenblum, "Improving precision by adjusting for prognostic baseline variables in randomized trials with binary outcomes, without regression model assumptions," *Contemporary clinical trials*, vol. 54, pp. 18–24, 2017.

[4] S. E. Robertson, J. A. Steingrimsson, and I. J. Dahabreh, "Using numerical methods to design simulations: revisiting the balancing intercept," *American journal of epidemiology*, vol. 191, no. 7, pp. 1283–1289, 2022.

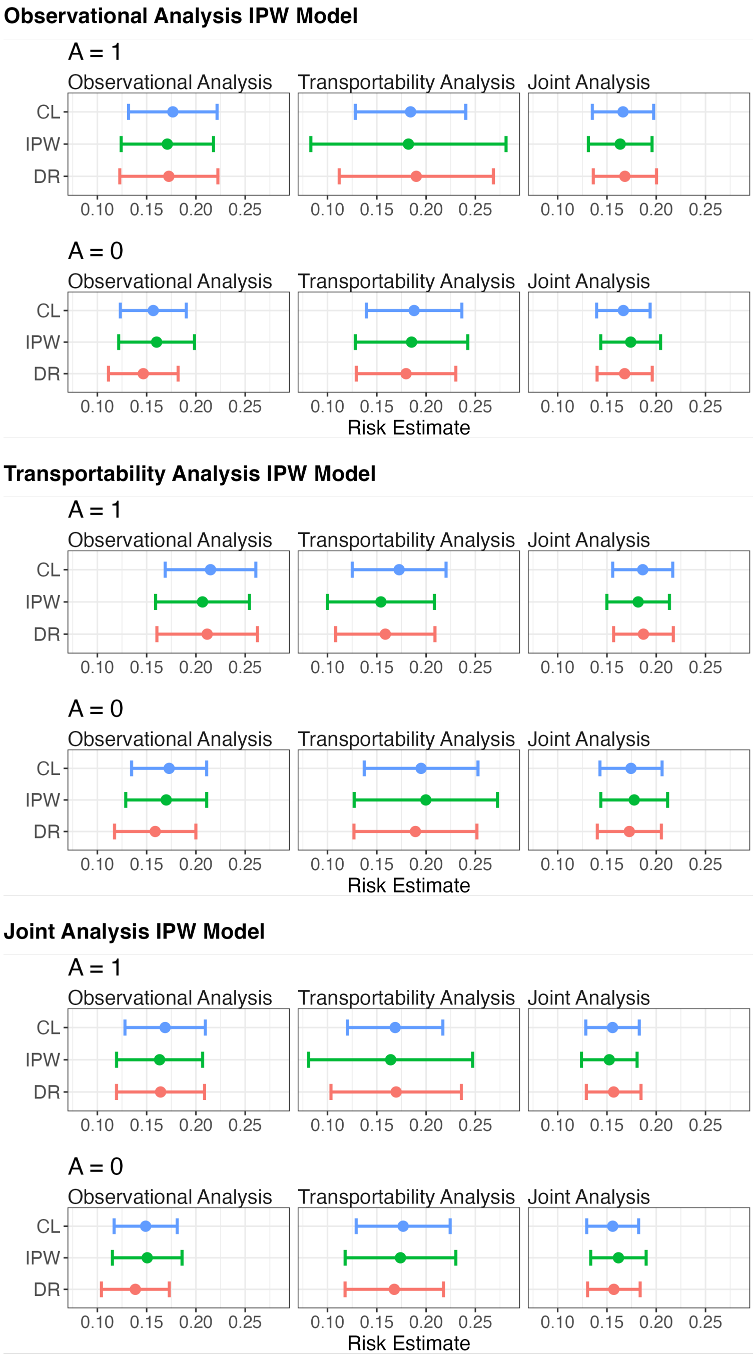

Figure 3: Counterfactual predictions for the three tailored models: Observational, Transportability and Joint Analysis (the inverse probability weighting strategy was used to fit the tailored model). Estimates and 95% confidence intervals of Brier risk in the population underlying the observational component of CASS for counterfactual treatments $A = 1$ (top) and $A = 0$ (bottom). Estimates are presented for the transportability, observational, and joint analysis. For each analysis, we present an outcome model (OM), inverse-weighting (IW) and doubly-robust (DR) estimators. 95% Wald confidence intervals were obtained using the non-parametric bootstrap with 500 bootstrap samples.

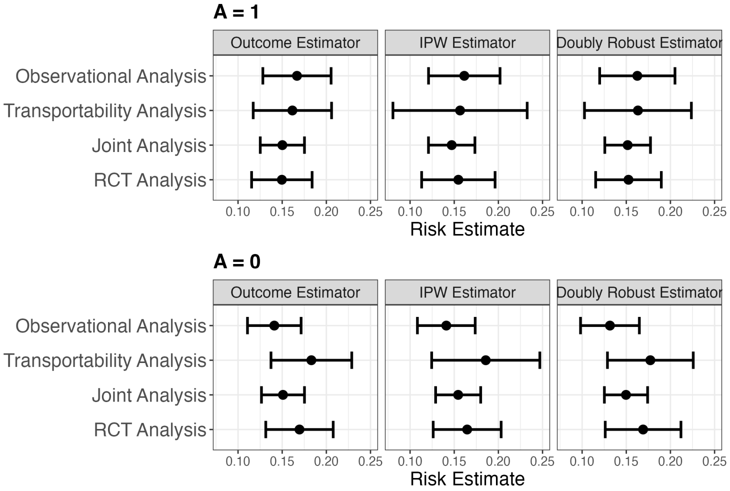

Figure 4: Estimates and 95% confidence intervals of Brier risk in the population underlying the observational component of CASS for counterfactual treatments $A = 1$ (top) and $A = 0$ (bottom). Estimates are presented for the transportability, observational, and joint analysis, as well as an additional analysis conducted on the randomized trial alone (RCT Analysis). For each analysis, we present an outcome model, inverse-probability-weighting (IPW) and doubly-robust estimators. 95% Wald confidence intervals were obtained using the non-parametric bootstrap with 500 bootstrap samples.
